# Seasonal Trends of Viral Prevalence and Incidence of Kawasaki Disease: A Korea Public Health Data Analysis

**DOI:** 10.3390/jcm10153301

**Published:** 2021-07-27

**Authors:** Jae Hee Lim, Yu Kyeong Kim, So Hyeon Min, Sang Won Kim, Young Hwan Lee, Jae Min Lee

**Affiliations:** 1 Department of Medicine, College of Medicine, Yeungnam University, Daegu 42415, Korea; imjh520@naver.com (J.H.L.); dbrud21206@naver.com (Y.K.K.); 3000min@naver.com (S.H.M.); 2 Medical Research Center, College of Medicine, Yeungnam University, Daegu 42415, Korea; kimsw3767@ynu.ac.kr; 3 Department of Pediatrics, College of Medicine, Yeungnam University, Daegu 42415, Korea

**Keywords:** Kawasaki disease, virus, children

## Abstract

Kawasaki disease (KD) is a systemic vasculitis that occurs mainly in children under 5 years of age and is often accompanied by coronary artery lesions. The cause of the disease remains undetermined, but it is estimated to result from viral or bacterial infections. Certain studies have shown infection as a leading cause of KD. The purpose of this study was to investigate the relationship between KD incidence and viral infections in different pediatric age groups, using the Health Insurance Review and Assessment (HIRA) Open Access Big Data Platform, to confirm seasonal trends by analyzing monthly patterns. We investigated the HIRA data of KD patients (M30.3) who were treated with intravenous immunoglobulin from 2015 to 2018. Weekly virus positive detection rate data (PDR) for this period was obtained from the Korea Disease Control and Prevention Agency for human adenovirus (HAdV), human parainfluenza virus (HPIV), human respiratory syncytial virus (HRSV), influenza virus (IFV), human coronavirus (HCoV), human rhinovirus (HRV), human bocavirus (HBoV), human metapneumovirus (HMPV), rotavirus, norovirus, and astrovirus. We then analyzed the weekly/monthly virus PDR and its association with KD incidence, including monthly incidence patterns, and seasonal trends. Seasonal trend analysis of the virus PDR was performed using the time series analysis method through ARIMA (Autoregressive Integrated Moving Average). Correlations between KD incidence and PDR at 1- and 2-month intervals were analyzed using the Granger test. A total of 16,740 patients were diagnosed with KD during the study period, mainly young children, with a male-to-female ratio of 1.35. Specifically, 15,635 (93%) patients were under 5 years of age, with an incidence rate of 172.4/100,000 person-years. Annually, the cumulative number of cases per month was the highest in January, with an average of 469 cases, and was the lowest in September, with an average of 291 cases, although most were diagnosed with KD in winter (29.3%). Granger tests showed that PDR for HRSV, rotavirus, and norovirus were related with KD incidence by 1 month, while PDR for HRSV, HRV, rotavirus, and norovirus by 2 months. This study found that detection rates of respiratory and enteric viruses preceded KD by 1–2 months. Further research is needed to confirm the association between these viruses and KD.

## 1. Introduction

Kawasaki disease (KD) is a systemic vasculitis that occurs mainly in children under 5 years of age, and is often accompanied by coronary artery lesions. It was first described by Tomisaku Kawasaki in 1967, and is now the most common cause of acquired heart disease in children in developed countries [1]. For KD diagnosis, a fever lasting more than 5 days, with findings of more than five characteristic clinical symptoms are required, in the setting where no other diagnosis could explain the results. The five characteristic clinical symptoms of KD are (1) bilateral conjunctival injection; (2) oral mucosal changes; (3) polymorphous skin rash; (4) peripheral extremity changes; and (5) cervical lymphadenopathy [2,3]. Incomplete KD may be considered in the presence of only one or two principal clinical features after very careful, sufficient observation, and excluding other diagnoses. Although the incidence rate varies among countries, Asian countries are expected to have a higher KD incidence than most Western countries [4].

The cause of KD has yet to be identified, but it is estimated to be caused by infectious diseases, such as viruses or hemolytic streptococcus, given that several studies have confirmed this association with common respiratory viruses. However, the role of respiratory viruses in KD pathogenesis remains unknown, and studies on this association with various viruses other than respiratory viruses are insufficient [5,6].

Thus, we aimed to analyze public health data provided by the Health Insurance Review and Assessment (HIRA) Open Access Big Data Platform and Korea Disease Control and Prevention Agency (KDCA) to determine the relationship between respiratory and gastrointestinal viruses and KD incidence.

## 2. Materials and Methods

### 2.1. Study Population

**Figure 1:**
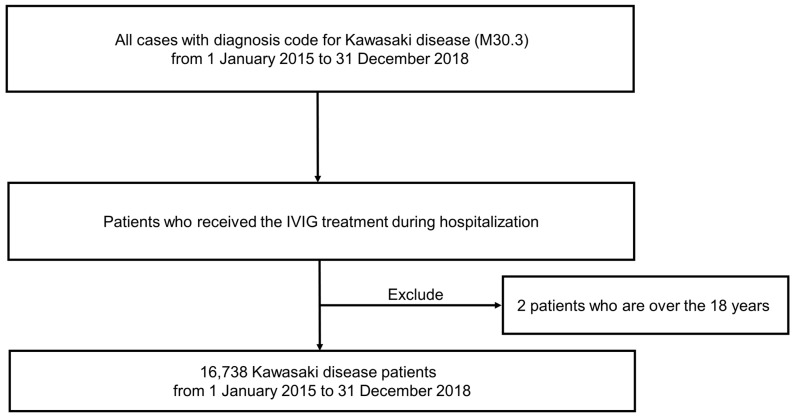
Flowchart illustrating patient selection.

We extracted data regarding KD from the HIRA, which is a government-affiliated organization created to build an accurate claims review and quality assessment system for the National Health Insurance, with databases open for all academic investigators [7,8,9]. Claims data in the HIRA database include patient diagnosis, treatment, procedures, surgical history, and prescription drugs, serving as a valuable resource for healthcare service research. We studied the HIRA data of KD patients who received IVIG treatment (ICD-10, M30.3), including a total of 16,740 incident KD cases between 1 January 2015, and 31 December 2018 (Figure 1). Most patients were under 18 years of age, with only two patients older than 18 years. Thus, only 16,738 KD patients were analyzed, excluding the two patients aged >18 years.

### 2.2. The Surveillance Data of Virus

We used the data reported by the KDCA on viruses that cause acute respiratory infections and gastroenteritis, wherein more than 4000 respiratory and 2000 enteric specimens were collected from 17 local environmental and health institutes, and over 100 participating hospitals across Korea during each year of the study period. The causative pathogens were then identified using standardized diagnostic procedures in a central laboratory, in which pathogen prevalence was surveyed weekly, and was analyzed based on genetic testing of influenza-like illness or acute diarrhea patients. The positive detection rate (PDR) data was then collected during the study period from 2015 to 2018, calculating the average monthly PDRs of seven respiratory viruses (adenovirus (HAdV), parainfluenza virus (HPIV), respiratory syncytial virus (HRSV), influenza virus (IFV), coronavirus (HCoV), rhinovirus (HRV), and bocavirus (HBoV)) and four acute diarrhea viruses (adenovirus, rotavirus, norovirus, and astrovirus).

### 2.3. Statistical Analyses

For incidence rate calculations, we used the 2015 Korean population data edited by the Ministry of the Interior and Safety as the denominator, with a total of 8,961,805 people under the age of 18 years. We then constructed a model of variations in ITP diagnosis, using the autoregressive integrated moving average (ARIMA) modeling approach, which assumes that the current observation is related to past observations over time. The general multiplicative form of the ARIMA model was denoted as (p, d, q), wherein p, d, and q were the order values of non-seasonal, autoregressive, differencing, and moving average parameters, respectively. Additionally, the autocorrelation function (ACF) was examined to identify the general form of the model to be fit. Considering the ACF graphs, different ARIMA models were identified for model selection (Supplemental Figure S1), and the minimum Akaike’s information criterion model was chosen as the best-fit model (Supplemental Table S1). Moreover, the Granger approach was used to investigate how many of the current values in the time series y could be described as other values [10]. The data were analyzed using the R software, and significance was defined as *p* < 0.05 for all analyses.

## 3. Results

### 3.1. Patient Characteristics

During the 4-year-period, 16,740 patients were diagnosed with KD (Table 1). Of these, 15,635 (93.4%) patients were under 5 years of age, 6993 (41.8%) were aged 0–1 years, 8642 (51%) were aged 1.1–5 years, 1103 (6.6%) were aged 5.1–18 years, and only 2 (0.0%) were over 18 years of age. Regarding sex, there were 9631 male and 7109 female patients in the study. The KD incidence rate for patients under 18 years of age was 46.7/100,000 person-years (Table 2). Based on age group, the incidence rates were 172.4, 411.8, 117.3, and 4.1 in 0–5 years, 0–1 years, 1.1–5 years, and 5.1–18 years, respectively.

### 3.2. Trend Analysis of KD

**Figure 2:**
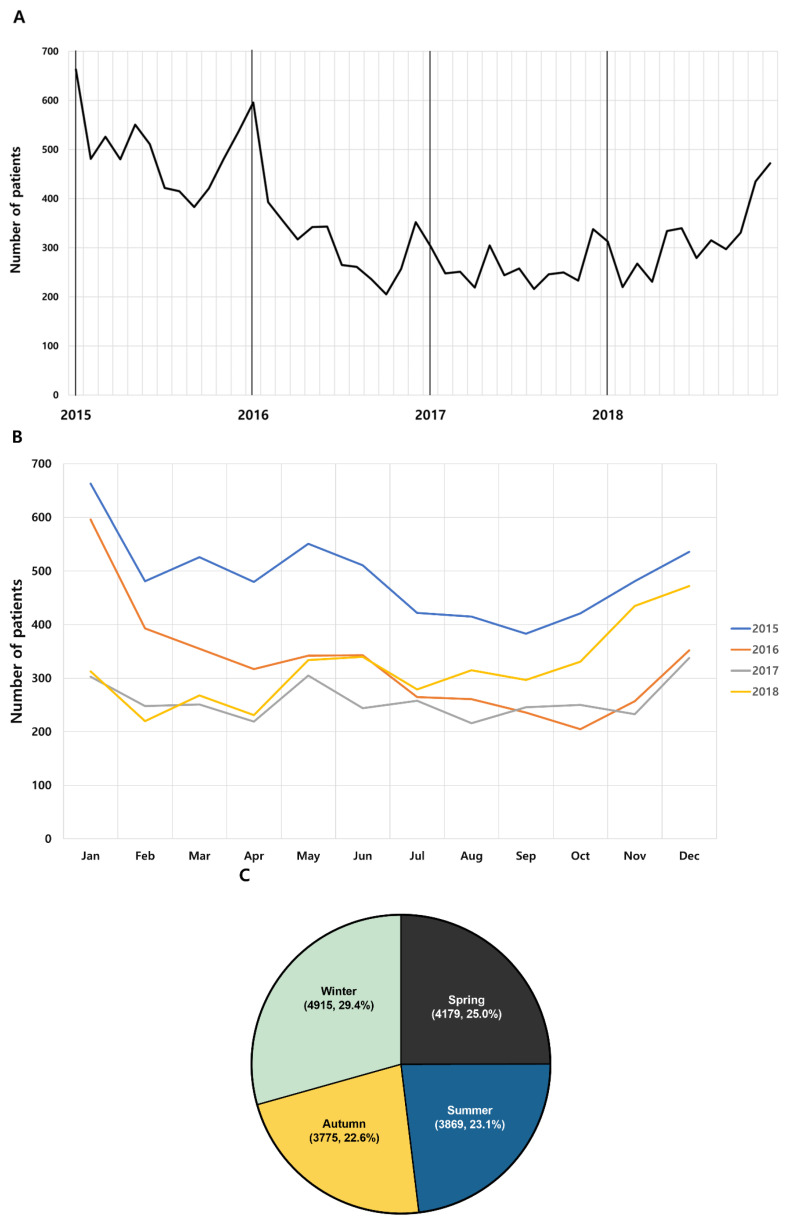
Incidence trend of Kawasaki disease from 2015 to 2018. (**A**) Monthly trend analysis Kawasaki disease from 2015 to 2018. (**B**) Monthly trend analysis of Kawasaki disease according to year. (**C**) Seasonal trend Kawasaki disease incidence. Spring (March to May), summer (June to August), autumn (September to November) and winter (December to February). (Cumulative incidence for 4 years, %).

Of the 16,738 patients under 18 years of age, 5870 were diagnosed in 2015, 3922 in 2016, 3111 in 2017, and 3835 in 2018. The highest incidence rate in 2015–2016 was in January, and in 2017–2018, it was highest in December (Table 3). Overall, the cumulative cases per month for four years were found to be the highest in January and the lowest in September (Figure 2A,B). Additionally, KD patients were most often diagnosed during winter (29.4%), followed by spring (25.0%), summer (23.1%), and then autumn (22.6%) (Figure 2C). The average number of cases per month was 348.7, and the average number of cases per year between 2015 and 2018 was 4184.5.

### 3.3. Positive Detection Rates of Virus

The PDRs of most viruses showed seasonal variations (Supplemental Table S2). Specifically, HAdV was highest in November, HPIV was highest in May, and HRSV was especially high in winter, with the highest number of HRSV cases in November. Furthermore, IFV was highest from November to February, and HCoV and norovirus were the highest from November to January. HRV and enteric HAdV were highest in September; HBoV was highest from May to June; and HMPV, rotavirus, and astrovirus were the highest in April, March, and January, respectively. The PDR of all viruses was highest in December, and lowest in July.

### 3.4. Causality between KD and Virus Prevalence

**Figure 3:**
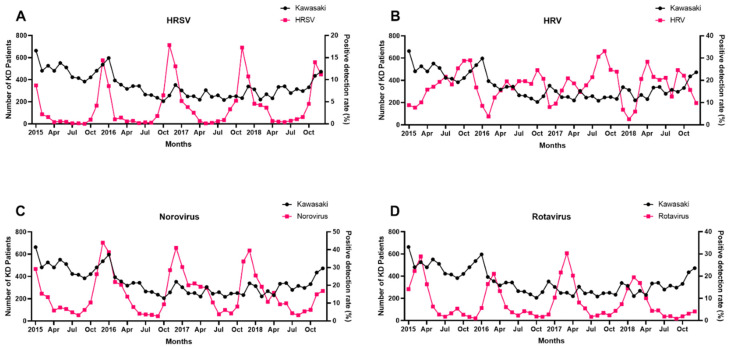
Relationship between PDR of (**A**) HRSV, (**B**) HRV, (**C**) norovirus, (**D**) rotavirus and incidence of KD during the study period.

If any prevalent virus affected KD diagnosis, the prevalence of that virus might increase before the peak of KD diagnosis. Thus, a Granger causality test was conducted between the virus PDR and KD diagnostic data collected 1 to 2 months later. The results of this test are shown in Table 4. Among the seven respiratory and four gastrointestinal viruses, the prevalence of certain viruses increased 1–2 months before the KD incidence increased. The PDRs for HRSV (*p <* 0.001), rotavirus (*p* = 0.048), and norovirus (*p <* 0.001) in patients aged <5 years correlated with increased KD incidence after 1 month (Table 4A); however, there was no statistical correlation between KD incidence and patients aged 5–18 years, and rotavirus was not statistically correlated at 1–5 years of age. Moreover, the PDRs for HRSV (*p <* 0.001), HRV (*p* = 0.001), rotavirus (*p* = 0.032), and norovirus (*p <* 0.001) in patients aged <5 years correlated with increased KD incidence after 2 months (Table 4B). There was no statistical correlation between KD incidence and patients aged 5–18 years, and rotavirus was also not statistically correlated at 1–5 years of age. Figure 3 shows the relationship between the PDRs of HRSV, HRV, norovirus, rotavirus, and KD incidence during the study period.

## 4. Discussion

This study evaluated the incidence of KD and its association with viral infections. We found that certain respiratory and gastroenteritis viral PDR were significantly associated with KD incidence after 1 and 2 months.

The KD incidence in Northeast Asian countries, such as South Korea, Japan, China, and Taiwan, is 10–30 times higher as compared to Western countries, such as the United States and Europe [11,12,13]. In a 2014 Japanese nationwide study, the number of KD patients was reported to be 15,979, resulting in an annual incidence rate of 308.0 per 100,000 children aged 0–4 years, with a M/F ratio of 1.32. This was the highest recorded KD incidence in Japan [12]. In a previous Korean study analyzed using the HIRA database, a total of 39,082 patients was reported between 2007 and 2014 [14]. The M/F ratio was 1.42, and the median age was 28 months. The incidence rates were 210.4 per 100,000 children aged 0 to 4 years in 2013 and 217.2 in 2014.

In this study, KD incidence based on age and sex was similar to the previous Korean study. A total of 16,740 KD patients were diagnosed between 2015 and 2018, with a M/F ratio of 1.36 and a median age of 30 months. Of these, 93.4% were under 5 years of age, and 41.8% were aged 0–1 years. The incidence rate was 172.4/100,000 children under 5 years of age, 411.8 in 0–1 years, and 117.3 in 1–5 years of age.

It is generally established that KD may be triggered by an infectious agent which activates the immune system in a genetically susceptible host [4,15,16]. As a basis for this, seasonal clustering in winter and spring is similarly seen in other viral diseases. Furthermore, temporal clusters of epidemics have already been reported in several countries [13].

Although KD had temporal clustering, different seasonality was found in different countries. In this study, the prevalence of KD in Korea was high in spring and winter, as in previous studies [14]. In Europe, the prevalence is high in winter, and in China, the prevalence is high in spring and summer [17,18]. In Japan, the prevalence is high in January and summer [19]. No single pathogen associated with KD has been reported worldwide. Chang et al. argued that it is not the same infectious etiology that causes KD, but is a heterogeneous source of infection according to regions and seasons [4].

KD is most common in children between 6 months and 5 years of age, making them the most susceptible to infections, before improving 1–3 weeks after onset [20]. In the Japanese nationwide survey, KD showed a peak incidence in January and February in winter [21], which is similar to our study given that Korean KD also occurred most frequently in winter (29.4%).

Many attempts have also been made to elucidate the association between viruses (adenovirus, rhinovirus, bocavirus, coronavirus, CMV, EBV, herpes, poliovirus, rotavirus) and KD, and while some have obtained meaningful data, no clear answer has yet been obtained [4,22,23,24,25,26].

In this study, we found that the HRV and HRSV prevalence among other respiratory viruses and KD incidence were statistically correlated.

Notably, HRV is relatively recent, making its association with KD more probable. Chang et al. conducted a prospective case-control study on 226 KD children and confirmed the association between various viruses, including HRV and KD [4]. Additionally, Turnier et al. retrospectively analyzed 192 KD patients, wherein 41.9% of the patients tested positive for respiratory virus PCR, with most of them positive for rhinovirus/enterovirus [5]. Moreover, L’Huillier et al. confirmed KD pediatric blood using high-throughput sequencing. As a result, it was reported that there was a temporal association with KD using vaccine records, such as polio and measles, hinting possible associations with rhinovirus and bocavirus [27]. These studies are consistent with our findings and can be considered as a reinforcement of our results.

Regarding HRSV, its association with KD appears to be low, given the lacking evidence in several studies attempting to establish an association with KD in all respiratory viruses [23]. In fact, only one case of co-infection of HRSV and HMPV in a 24-month-old child with KD was reported [28]. This is the first study to check the association between HRSV and KD using national health big data.

In comparison, many studies have investigated the association between HAdV and KD [22,29,30,31,32]. Song et al. reported that the adenovirus is very common and is similar to KD in that it has the highest disease incidence in children under 5 years of age. In addition, the majority of children with adenovirus had clinical characteristics similar to those of KD in that study. Based on this, they were able to accidentally detect adenovirus in KD patients; thus, KD diagnosis cannot be completely excluded when an adenovirus is detected [22]. In our study, there was no significant difference between adenovirus prevalence and KD incidence under 18 years of age, which may be related to the limitations of our study.

Compared to respiratory viruses, few studies have been conducted on the association between gastrointestinal viruses and KD, showing an association between KD and rotavirus infection. A study by Matsuno et al. presented evidence of rotavirus infection in KD patients, wherein 39 samples were obtained from hospitalized KD patients, and rotavirus capsomer aggregates were found in 26 of them [26]. Mellone et al. also reported that monovalent and pentavalent rotavirus vaccines lowered KD incidence [33], although some cases have reported KD following rotavirus vaccination [34]. In addition, a cohort study using commercial insurance data investigated the side effects associated with the rotavirus vaccine, showing that 23 cases were diagnosed with KD out of a total 2,468,002 concomitant diphtheria–tetanus–pertussis vaccinations among children [35].

In this study, the rotavirus was statistically associated for children under the age of 1 year; however, no association was found for children aged 1–5 years, suggesting that extensive rotavirus vaccination may have affected the results. The rotavirus vaccine in South Korea is a 2- or 3-dose schedule indicated for infants aged 6–32 weeks (i.e., at 2 and 4 months, or at 2, 4, and 6 months) [36]. Moreover, rotavirus antibodies are detected in the blood serum within 2 days after diarrhea onset [37]. In that regard, it is assumed that the antibody formation of rotavirus may have influenced KD incidence.

In our current study, the PDR of norovirus was also found to be related to KD incidence. One case reported that secondary infections, such as norovirus infection, can worsen systemic vasculitis, such as Henoch–Schonlein purpura [38]. However, it is not yet known how norovirus infection affects KD development. Research on this topic is needed in future studies.

Despite these findings, this study had certain limitations. First, this was a retrospective study, which has a lower evidence level when compared to prospective studies and may have had selection bias. Second, as a limitation of data, the information collected from HIRA did not include the clinical content of the patients. Moreover, patients who had been tested for viral infection were different from those who had been diagnosed with KD. Therefore, it was not possible to establish a direct relationship between viral infection and KD, since only the predecessor relationship could be assessed through the incidence rate. However, the trend of incidence with virus detection rate in KD was represented by a time series graph and was analyzed using the Granger causality test. The association between the number of KD patients and viral transmission cannot be definitively concluded. Since viral infections are common in winter, much more epidemiological data is needed to link viral epidemics to the onset of Kawasaki disease.

## 5. Conclusions

This study evaluated KD incidence and investigated its correlation with the prevalence of common viral respiratory and gastrointestinal infections. To our knowledge, and to date, this is the largest nationwide analysis of patients with KD and their association with the PDRs of common viruses in Korea. In Korea, infection rates by HRSV, rotavirus, norovirus, and HRV were found to precede the incidence of KD by 1–2 months. Thus, it is possible that these viruses acted as triggers for KD development. Prospective studies on these respiratory and gastrointestinal viruses and KD are needed in the future to further elucidate these associations.

## Figures and Tables

**Table 1:** Characteristics of patients.

Variables		*n* (%)
Total number of patients		16,740 (100.0)
Age group		
	0–5 years	15,635 (93.4)
	0–1 years	6993 (41.8)
	1.1–5 years	8642 (51.6)
	5.1–18 years	1103 (6.6)
	>18 years	2 (0.0)
Sex		
	Male	9631 (57.5)
	Female	7109 (42.5)
Location		
	Seoul	3874 (23.1)
	Busan	834 (5.0)
	Daegu	1206 (7.2)
	Incheon	1196 (7.1)
	Gwangju	536 (3.2)
	Daejeon	822 (4.9)
	Ulsan	361 (2.2)
	other	7911 (47.4)
Insurance type		
	Medical insurance	16,623 (99.3)
	Medical aid	117 (0.7)
	Other	

**Table 2:** Characteristics of patients under 18 years of age.

Age Group (Years)	Number of Patients, *n* (%)	Male, *n* (%)	Female, *n* (%)	M:F Ratio *	Incidence Rate **
0.1–5	15,635 (93.4)	9009 (53.5)	6626 (39.3)	1.36	172.4
0.1–1	6993 (41.8)	4197 (25.0)	2796 (16.6)	1.5	411.8
1.1–5	8642 (51.6)	4812 (28.6)	3830 (22.7)	1.26	117.3
5.1–18	1103 (6.6)	621 (3.7)	482 (2.9)	1.29	4.1
Total	16,738 (100.0)	9630 (57.5)	7108 (42.5)	1.35	46.7

* M:F ratio = male-to-female ratio. ** Incidence rate per 100,000 population.

**Table 3:** Monthly numbers of newly diagnosed pediatric Kawasaki disease patients in Korea.

	January	February	March	April	May	June	July	August	September	October	November	December	Total	Average
2015	663	481	526	480	551	511	422	415	383	421	481	536	5870	489.2
2016	596	393	355	317	342	343	265	261	236	205	257	352	3922	326.8
2017	303	248	251	219	305	244	258	216	246	250	233	338	3111	259.3
2018	313	220	268	231	334	340	279	315	297	331	435	472	3835	319.6
Total	1875	1342	1400	1247	1532	1438	1224	1207	1162	1207	1406	1698	16,738	1394.8
Average	468.8	335.5	350.0	311.8	383.0	359.5	306.0	301.8	290.5	301.8	351.5	424.5	4184.5	348.7

**Table 4:** Values of the Granger causality test between the time series of Kawasaki disease diagnosis and the time points of positive detection rates of virus, with <0.05 indicating significance (written in bold).

**A. Diagnostic Data and Virus Data after 1 Month**												
**Age Group (Years)**	**HAdV**	**HPIV**	**HRSV**	**IFV**	**HCoV**	**HRV**	**HBoV**	**HMPV**	**Rotavirus**	**Norovirus**	**Adenovirus**	**Astrovirus**
<5	0.720	0.495	**<0.001**	0.271	0.221	0.279	0.478	0.236	**0.048**	**<0.001**	0.505	0.586
0–1	0.756	0.593	**<0.001**	0.216	0.102	0.672	0.644	0.850	**0.014**	**<0.001**	0.519	0.459
1.1–5	0.552	0.392	**<0.001**	0.516	0.403	0.259	0.298	0.059	0.256	**0.007**	0.612	0.887
5.1–18	0.970	0.532	0.210	0.661	0.389	0.220	0.476	0.567	0.590	0.290	0.636	0.783
Total	0.721	0.487	**<0.001**	0.337	0.289	0.256	0.438	0.294	0.053	**<0.001**	0.432	0.662
**B. Diagnostic Data and Virus Data after 2 Months**												
**Age Group (Years)**	**HAdV**	**HPIV**	**HRSV**	**IFV**	**HCoV**	**HRV**	**HBoV**	**HMPV**	**Rotavirus**	**Norovirus**	**Adenovirus**	**Astrovirus**
<5	0.129	0.603	**<0.001**	0.368	0.190	**0.001**	0.448	0.541	**0.032**	**<0.001**	0.668	0.859
0–1	0.181	0.936	**<0.001**	0.340	0.138	**0.031**	0.871	0.991	**0.046**	**<0.001**	0.448	0.764
1.1–5	0.145	0.352	**0.030**	0.659	0.374	**0.002**	0.219	0.185	0.076	**0.017**	0.911	0.993
5.1–18	0.657	0.812	0.111	0.717	0.194	0.307	0.155	0.661	0.165	0.243	0.096	0.625
Total	0.151	0.591	**<0.001**	0.426	0.183	**0.002**	0.382	0.594	**0.028**	**<0.001**	0.636	0.916

HAdV, human adenovirus; HPIV, human parainfluenza virus; HRSV, human respiratory syncytial virus; IFV, influenza virus; HCoV, human coronavirus; HRV, human rhinovirus; HBoV, human bocavirus; HMPV, human metapneumovirus.

## Data Availability

The data presented in this study are available on request from the corresponding author.

## Supplementary Materials

The following are available online at https://www.mdpi.com/article/10.3390/jcm10153301/s1, Table S1: Parameters of ARIMA models for Kawasaki disease patients by age, Table S2: Positive detection rates of virus during study period, Figure S1: Residual ACF correlogram and 95% confidence limits for newly diagnosed Kawasaki disease.
