# Supplementary material for: Seasonal Trends of Viral Prevalence and Incidence of Kawasaki Disease: A Korea Public Health Data Analysis
Source: J Clin Med. 2021 Jul 27;10(15):3301. doi: 10.3390/jcm10153301 (PMC8347058; doi:10.3390/jcm10153301)
Supplement: Supplementary file 1 [file jcm-10-03301-s001.zip › jcm-1251849-supplementary.pdf]

Supplementary Materials

Table S1. Parameters of ARIMA models for Kawasaki disease patients by age.

Parameters	0–1 years	1.1–5 years	0–5 years	5.1–18 years	Total
p autoregressive	0	0	0	0	0
d difference	1	1	1	1	1
q moving average	0	0	0	1	0
AIC	473.1	464.81	521.71	318.12	528.69

AIC, Akaike's Information Criterion.

Table S2. Positive detection rates of virus during study period.

Mon PDR(%)	Jan	Feb	Mar	Apr	May	Jun	Jul	Aug	Sep	Oct	Nov	Dec
**HAdV**												
2015	5.04	2.15	2.83	4.70	4.83	5.63	3.32	3.18	2.30	6.98	9.03	8.78
2016	9.18	3.88	3.93	5.54	6.53	6.95	7.16	9.48	9.58	6.50	5.83	3.24
2017	3.72	2.53	2.43	2.74	6.05	4.73	2.96	5.18	4.33	3.78	4.73	2.60
2018	2.98	2.80	4.25	5.38	5.45	6.80	5.82	9.55	13.33	11.30	10.70	7.63
**HPIV**												
2015	0.92	0.55	0.78	6.30	17.65	11.75	7.04	6.28	8.93	4.72	9.53	4.08
2016	2.24	1.30	1.40	5.60	16.55	16.23	9.68	10.25	8.68	4.56	1.95	0.78
2017	1.58	1.70	2.98	8.38	19.43	12.83	9.68	8.95	4.60	3.04	3.33	2.68
2018	0.84	1.60	3.25	9.44	19.10	16.78	11.12	7.75	4.33	4.00	1.75	1.03
**HRSV**												
2015	8.70	2.15	1.55	0.40	0.58	0.45	0.12	0.13	0.00	0.96	4.13	14.40
2016	8.56	1.00	1.43	0.56	0.70	0.15	0.34	0.28	1.78	6.46	17.83	13.02
2017	5.16	3.83	2.53	0.64	0.10	0.23	0.60	0.85	3.30	5.24	17.25	10.73
2018	4.58	4.25	3.65	0.64	0.50	0.40	0.72	1.05	1.55	4.52	13.95	11.20
**IFV**												
2015	22.80	48.85	45.28	25.96	3.10	0.33	0.14	0.00	0.38	0.10	0.48	1.53
2016	15.24	47.55	36.95	26.60	5.00	0.70	0.22	0.28	0.25	0.40	0.80	35.50
2017	26.20	7.70	8.95	12.72	5.03	2.20	1.10	0.55	0.93	0.66	4.05	41.55
2018	58.50	35.63	10.55	3.46	2.80	1.08	0.50	0.30	0.58	1.78	11.15	35.35
**HCoV**												
2015	8.74	1.40	0.95	0.56	0.10	0.50	0.58	0.60	0.38	1.38	1.80	5.53
2016	7.96	3.98	4.95	4.26	4.90	3.08	3.94	4.53	4.68	4.24	7.80	9.82
2017	10.26	6.45	5.15	3.62	2.68	2.35	1.02	1.45	1.55	2.14	5.15	7.20
2018	6.76	9.88	7.73	3.52	0.85	0.48	1.00	3.13	2.60	5.80	10.93	9.83
**HRV**												
2015	8.80	7.73	10.08	15.84	17.10	19.30	21.62	18.10	25.40	28.82	29.03	16.78
2016	8.52	3.78	12.28	15.44	19.55	16.38	19.62	19.60	18.40	24.56	20.68	8.02
2017	9.56	15.40	20.90	18.66	14.78	17.78	21.40	30.63	33.10	24.68	23.88	6.83
2018	2.52	5.95	20.63	28.34	21.53	20.18	21.14	12.70	24.63	22.08	15.70	9.78
**HBoV**												
2015	0.58	0.70	0.68	5.18	9.05	8.18	2.46	0.18	0.00	0.56	0.23	0.55
2016	1.06	0.83	1.93	3.86	4.55	2.80	1.06	1.15	0.83	0.30	0.25	0.30
2017	0.58	1.00	1.35	4.66	9.33	3.98	0.82	0.30	0.25	0.18	0.20	0.58
2018	0.26	0.70	0.10	0.88	4.35	7.18	5.78	0.55	0.63	0.60	0.85	0.88
**HMPV**												
2015	0.66	0.40	0.98	3.64	7.10	4.20	1.48	0.33	0.13	0.46	0.43	1.68
2016	3.56	4.20	8.05	12.64	8.53	2.83	0.66	1.20	0.98	0.42	1.05	1.14
2017	4.76	11.10	16.38	14.14	4.80	0.63	0.56	0.10	0.00	0.76	0.60	0.28
2018	0.86	2.95	8.60	19.12	15.33	6.00	2.82	0.73	0.48	0.08	0.18	0.15
**Group A Rotavirus**												
2015	14.10	22.35	28.90	16.38	6.33	2.70	1.72	3.25	5.38	2.62	1.65	0.98
2016	5.66	16.45	21.08	13.28	6.03	3.73	2.30	4.20	3.43	1.86	1.68	2.76
2017	10.38	21.68	30.33	20.26	8.13	5.53	1.68	2.30	3.45	2.34	4.35	7.38
2018	14.52	19.53	16.88	10.10	4.38	4.55	1.84	1.98	0.85	1.90	3.13	4.10
**Norovirus**												
2015	29.20	15.30	13.35	5.82	7.53	6.73	4.86	3.20	6.18	10.36	26.28	43.98
2016	38.62	21.85	20.33	13.64	7.80	4.08	3.66	3.40	2.65	9.32	28.58	41.06
2017	30.28	20.00	21.08	19.18	18.58	10.40	3.72	6.20	4.23	8.06	33.40	39.58
2018	25.46	19.20	10.78	15.74	9.30	9.90	4.34	3.20	5.35	6.28	14.95	16.90
**Enteric Adenovirus**												
2015	1.68	3.03	1.55	1.70	2.30	1.05	0.28	0.00	0.60	2.22	0.55	1.80

2016	2.28	2.58	1.25	2.30	2.28	2.75	2.16	3.43	10.73	6.88	5.68	6.78
2017	2.10	2.23	0.55	2.86	2.90	3.05	2.78	3.30	3.50	5.04	3.43	1.13
2018	2.40	1.85	2.55	3.04	2.85	5.65	4.44	6.45	6.03	3.82	3.33	2.15
						**Astrovirus**						
2015	2.30	1.28	2.30	2.68	3.25	3.05	0.36	0.58	1.73	1.14	0.70	0.68
2016	0.94	1.18	2.48	3.20	3.40	4.30	2.52	2.28	3.43	2.82	2.05	0.86
2017	11.96	1.15	1.73	5.54	3.90	4.08	3.32	2.30	1.78	2.08	0.68	2.58
2018	0.94	1.73	0.00	1.82	3.05	2.50	2.74	5.18	5.63	3.98	2.73	2.33

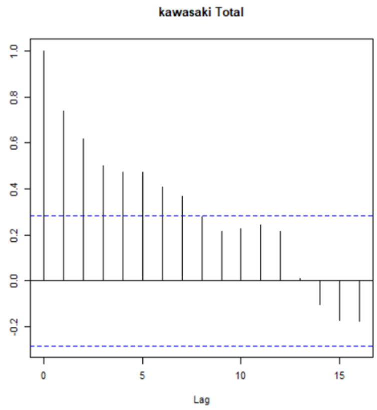

(**a**)

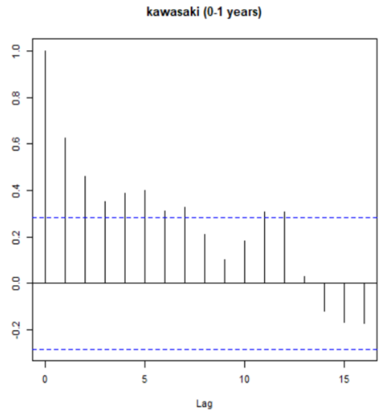

(**b**)

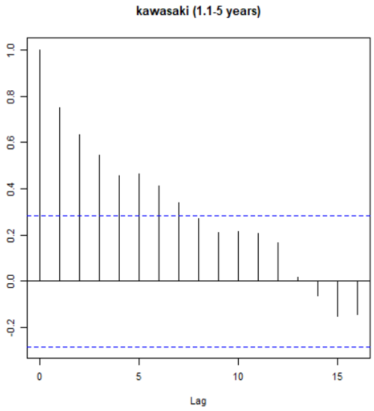

(**c**)

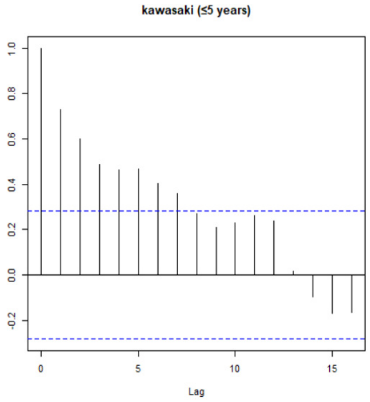

(**d**)

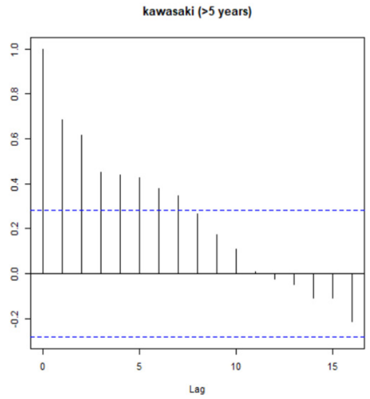

(**e**)

Figure S1. Residual ACF correlogram and 95% confidence limits for newly diagnosed Kawasaki disease.
